# Sir William Withey Gull (1816–1890)

**DOI:** 10.1007/s00415-016-8250-9

**Published:** 2016-08-08

**Authors:** Antoni Niedzielski, Natalia Kaźmierczak, Andrzej Grzybowski

**Affiliations:** 1 Faculty of Health Sciences, Medical University of Lublin, ul. Staszica 4, 20-081 Lublin, Poland; 2 Department of Ophthalmology, Poznań City Hospital, Poznań, Poland; 3 Department of Ophthalmology, University of Warmia and Mazury, Olsztyn, Poland

The year 2016 is the bicentenary of Sir William Withey Gull’s birth, a baronet and a court physician of the Prince of Wales and Queen Victoria, and also an outstanding clinician. Gull was the first to describe anorexia nervosa. He also conducted studies on the spinal cord and helped to expand the state of neurological knowledge on disorders such as paraplegia and syringomyelia [1]. He also contributed to medical science with regards to thyroid gland-related mental deficiency and glomerulonephritis.

**Fig. 1:**
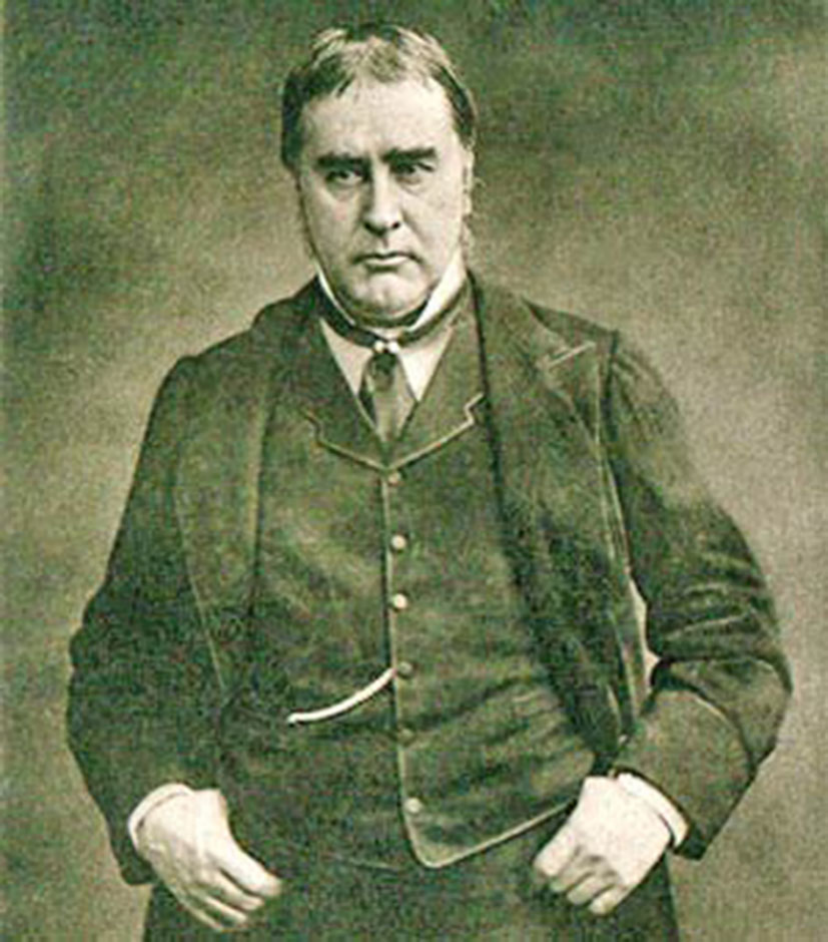
Sir William Withey Gull (1816–1890)

William Withey Gull was born on 31st December 1816 in Colchester, Essex [2, 3]. He was a son of John Gull and Elizabeth Gull. His father owned a barge named *The Dove*, on which William was born. In 1837 William Gull took his first job at Guy’s Hospital in London, where he also studied medical science. He was helped by B. Harrison, a local parish priest’s uncle who had taught William at an earlier stage of education. At this time William earned his reputation as a diligent and eminent student of medicine (Fig. 1).

In 1838 he passed his entrance exams to the University of London. Three years later he received his Bachelor of Medicine degree. In 1846 he became a physician and received a gold medal for his academic results. He lectured on physiology and comparative anatomy at Guy’s Hospital. He climbed the academic ladder receiving the title of Fullerian Professor of Physiology. In 1868 he received the PhD degree in civil law at Oxford University. In 1871–1883 he was a Crown member of the General Medical Council, and from 1886 he represented the University of London. In 1880 he received another PhD degree in law from both Cambridge and Edinburgh Universities.

As a court physician in 1871 he cured the Prince of Wales of typhoid fever. As a token of gratitude, he created a Baronet and appointed to be one of the Physicians-in-Ordinary to HM Queen Victoria. From 1887 he suffered the first of several strokes. He died on 29th January 1890 in his house at Brook Street in London. His death was reported in The Times. On 1st February 1890 Mark Twain wrote “Sir Wm. Gull is just dead. He nursed the Prince of Wales back to life in 1971 and apparently it was for this that Mr. Gull was granted a Knighthood, that doormat at the threshold of nobility. When the Prince seemed dead Mr. Gull dealt blow after blow between the shoulders, breathed into his nostrils, and literally cheated Death”. Gull’s body was buried next to the grave of his father and mother in the churchyard of his childhood home at Thorpe-le-Soken, near Colchester, Essex. A memorial bronze plaque was placed at the entrance to Guy’s Hospital Chapel.

In the nineteenth century, almost simultaneously, two physicians W.W. Gull and E. CH. Lasègue (1816–1883) described cases of anorexia [4, 5]. They both created a list of psychosomatic symptoms, however, they presented different etiologies for the disorder. Lasègue was the first to treat anorexia as a mental disorder, he used the term anorexia hysterique and attributed the disorder only to women. Gull termed the condition anorexia nervosa and believed that the disorder occurred in both male and female patients. The term anorexia nervosa entered the literature in 1873 [6]. It was then that Gull published his ground-breaking work with this title. Gull viewed anorexia from a medical point of view, believing that it was not of any organic etiology. He pointed to the observation that anorexia was mostly found in young girls, 16–23 years old, and its characteristic symptom was fatigue. Other symptoms were body mass loss, amenorrhoea, and general weakness. He also noticed that symptoms common to all the cases were slower breathing, lower pulse rate, and a fall in body temperature. He also observed that the patients were excessively active and full of energy even though they were skinny and did not meet their energetic demands. Gull saw the source of anorexia in “ego perversion”, and highlighted the importance of psychological factors behind the disorder.

Gull also took an interest in the functioning of the spinal cord [1, 7, 8] and distinguished three types of paraplegia: spinal, peripheral, and cerebral. He wrote his keynote work on paraplegia together with another physician, Charles-Édouard Brown-Séquard (1817–1894). The work was the first to clinically discuss the pathology of the spinal cord. He also described two types of damage, one limited to the segment of the spinal cord, and the other developing lengthwise, along its column. He was also interested in degeneration of the posterior columns of the spinal cord, which leads to inability to regulate the motor area. Gull claimed that leg paraplegia might be caused by disorders of the urinary bladder or kidneys (“urinary paraplegia”).

In 1873, Gull described cases of myxoedema and dementia in previously healthy women [9]. Gull was the first to state that the cause of myxoedema was atrophy of the thyroid gland. He described the symptoms and appearance changes that he observed in his patients. These included cessation of periods, sleepiness, increased body mass index, roundness of face, thickness of tongue, and guttural voice. A slight change in the mental state was observed. Those who were normally active, joyful and inquiring became sleepy and indifferent, however, their intellectual powers remained intact. Gull observed the skin became smooth and fair, and its delicate shade on the cheek was different from the one that can be observed on the face of the patient with kidney disorder. A few years later, in 1877, he introduced the term myxoedema [10].
